# 2,3-Dimethyl-6-nitro­quinoxaline

**DOI:** 10.1107/S1600536810024463

**Published:** 2010-06-26

**Authors:** Raza Murad Ghalib, Rokiah Hashim, Sayed Hasan Mehdi, Jia Hao Goh, Hoong-Kun Fun

**Affiliations:** a School of Industrial Technology, Universiti Sains Malaysia, 11800 USM, Penang, Malaysia; b X-ray Crystallography Unit, School of Physics, Universiti Sains Malaysia, 11800 USM, Penang, Malaysia

## Abstract

The asymmetric unit of the title quinoxaline compound, C_10_H_9_N_3_O_2_, contains two crystallographically independent mol­ecules (*A* and *B*). The quinoxaline ring systems are essentially planar, with maximum deviations of 0.006 (1) and 0.017 (1) Å, respectively, for mol­ecules *A* and *B*. In mol­ecule *A*, the dihedral angle formed between the quinoxaline ring system and nitro group is 10.94 (3)° [6.31 (13)° for mol­ecule *B*]. In the crystal, mol­ecules are linked into chains propagating along [001]: one forms zigzag chains linked by C—H⋯O hydrogen bonds, whilst the other forms ladder-like chains by way of C—H⋯N and C—H⋯O hydrogen bonds. The packing is further consolidated by weak π–π inter­actions [range of centroid–centroid distances = 3.5895 (7)–3.6324 (7) Å].

## Related literature

For general background to and applications of the title quinoxaline compound, see: Darabi *et al.* (2008 ▶). For the synthesis, see: Ajaikumar & Pandurangan (2009 ▶); Darabi *et al.* (2009 ▶). For related quinoxaline structures, see: Ghalib *et al.* (2010 ▶); Wozniak *et al.* (1993 ▶). For graph-set descriptions of hydrogen-bond ring motifs, see: Bernstein *et al.* (1995 ▶). For the stability of the temperature controller used for the data collection, see: Cosier & Glazer (1986 ▶).
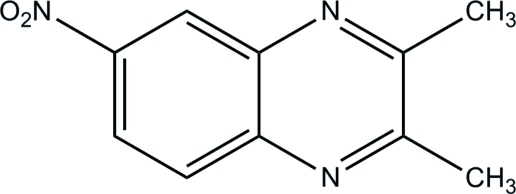


## Experimental

### 

#### Crystal data

C_10_H_9_N_3_O_2_
*M* *_r_* = 203.20
Monoclinic, 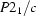

*a* = 7.1125 (7) Å
*b* = 22.490 (2) Å
*c* = 12.9596 (10) Å
β = 115.026 (4)°
*V* = 1878.4 (3) Å^3^
*Z* = 8
Mo *K*α radiation
μ = 0.10 mm^−1^
*T* = 100 K
0.26 × 0.21 × 0.10 mm

#### Data collection

Bruker APEXII DUO CCD diffractometer
Absorption correction: multi-scan (*SADABS*; Bruker, 2009 ▶) *T*_min_ = 0.973, *T*_max_ = 0.990
52279 measured reflections
7510 independent reflections
5559 reflections with *I* > 2σ(*I*)
*R*_int_ = 0.043

#### Refinement

*R*[*F* ^2^ > 2σ(*F* ^2^)] = 0.045
*wR*(*F* ^2^) = 0.136
*S* = 1.03
7510 reflections
275 parameters
H-atom parameters constrained
Δρ_max_ = 0.55 e Å^−3^
Δρ_min_ = −0.20 e Å^−3^

### 

Data collection: *APEX2* (Bruker, 2009 ▶); cell refinement: *SAINT* (Bruker, 2009 ▶); data reduction: *SAINT*; program(s) used to solve structure: *SHELXTL* (Sheldrick, 2008 ▶); program(s) used to refine structure: *SHELXTL*; molecular graphics: *SHELXTL*; software used to prepare material for publication: *SHELXTL* and *PLATON* (Spek, 2009 ▶).

## Supplementary Material

Crystal structure: contains datablocks global, I. DOI: 10.1107/S1600536810024463/hb5504sup1.cif

Structure factors: contains datablocks I. DOI: 10.1107/S1600536810024463/hb5504Isup2.hkl

Additional supplementary materials:  crystallographic information; 3D view; checkCIF report

## Figures and Tables

**Table 1:** Hydrogen-bond geometry (Å, °)

*D*—H⋯*A*	*D*—H	H⋯*A*	*D*⋯*A*	*D*—H⋯*A*
C3*A*—H3*A*⋯N2*A*^i^	0.93	2.56	3.4486 (14)	160
C9*B*—H9*D*⋯O1*B*^ii^	0.96	2.58	3.5380 (14)	176
C10*A*—H10*A*⋯O2*A*^i^	0.96	2.38	3.3355 (15)	171

Symmetry codes: (i) 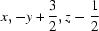; (ii) 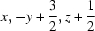.

**Fig. 1.**
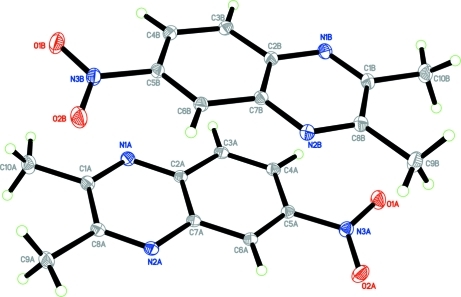
The molecular structure of (I) showing 30 % probability displacement ellipsoids for non-H atoms.

**Fig. 2.**
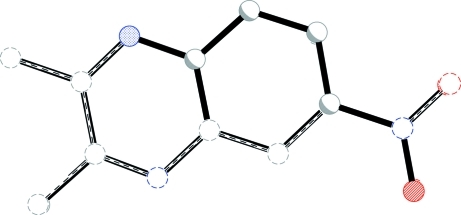
Fit of molecule A (dashed lines) on molecule B (solid lines). H atoms have been omitted for clarity.

**Fig. 3.**
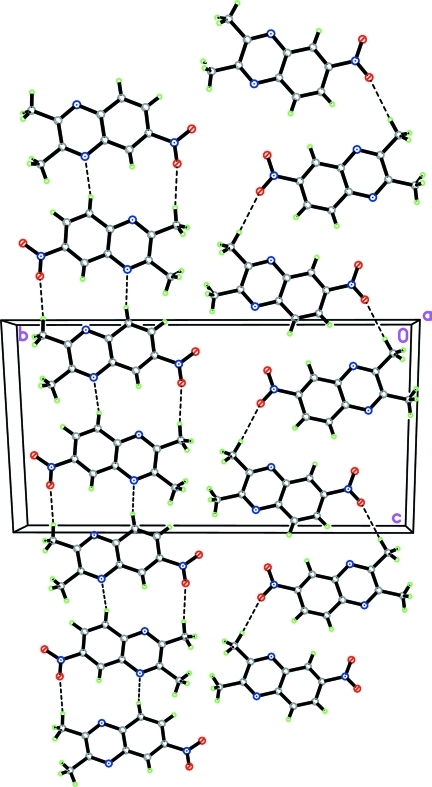
The crystal structure of (I), viewed along the a axis, showing the molecules being linked into one-dimensional chains along the [001] direction. Intermolecular hydrogen bonds are shown as dashed lines.

## supplementary crystallographic information

### Comment

The direct condensation of various benzene-1,2-diamines with 1,2-dicarboxyl compounds has been successfully achieved in excellent yields using (NH_4_Cl-CH_3_OH) catalyst system at room temperature (Darabi *et al.*, 2008). Here in this study our method comprises the synthesis of the title compound by the reaction of 4-nitro-o-phenylenediamine and butanedione in distilled water. The procedure can be performed for a broad scope of quinoxaline derivatives and is eco-friendly.

The asymmetric unit of the title quinoxaline compound comprises of two crystallographically independent 2,3-dimethyl-6-nitroquinoxaline molecules, designated molecules *A* and *B* (Fig. 1). The two independent molecules having closely similar geometries, as shown in the superposition of the non-H atoms of molecules *A* and *B* (Fig. 2) using *XP* in *SHELXTL* (Sheldrick, 2008), giving an r.m.s. deviation of 0.116 Å.

In each molecule, the quinoxaline ring system (C1-C8/N1/N2) is essentially planar, with maximum deviations of -0.006 (1) and -0.017 (1) Å, respectively, for atoms C1A of molecule *A* and C3B of molecule *B*. There are slight inclinations between the quinoxaline ring systems and nitro groups, as indicated by the dihedral angles formed of 10.94 (3) and 6.31 (13)°, respectively, for molecules *A* and *B*. The bond lengths and angles are comparable to those observed in the reported quinoxaline structures (Ghalib *et al.*, 2010; Wozniak *et al.*, 1993).

The interesting feature of the crystal packing (Fig. 3) is that no intermolecular hydrogen bond is observed between the two independent molecules and they are packed in different manners. Adjacent molecules *A* are linked by intermolecular C3A—H3A···N2A and C10A—H10A···O2A hydrogen bonds (Table 1) into ladder-like chains incorporating *R*^2^_2_(13) ring motifs (Bernstein *et al.*, 1995) whereas intermolecular C9B—H9D···O1B hydrogen bonds (Table 1) link adjacent molecules *B* into zig-zag shaped chains. Both chains are running along the [001] direction. Further consolidation of the crystal packing is provided by weak *Cg*1···*Cg*2 and *Cg*1···*Cg*3 interactions [*Cg*1···*Cg*2 = 3.5895 (7) Å, symmetry code: x, y, z; *Cg*1···*Cg*2 = 3.6324 (7) Å, symmetry code: x-1, y, z; *Cg*1···*Cg*3 = 3.6228 (7) Å, symmetry code: x, y, z; *Cg*1 and *Cg*2 are the centroids of the C2A–C7A and C2B–C7B benzene rings, respectively; *Cg*3 is the centroid of the C1B/N1B/C2B/C7B/N2B/C8B pyrazine ring].

### Experimental

The title compound was synthesized as reported in the literatures (Darabi *et al.*, 2009; Ajaikumar & Pandurangan, 2009). A mixture of 4-nitro-o-phenylenediamine (1.5310 g) and butanedione (0.8775 g) in molar ratio 1:1 were refluxed in distilled water for 1 h. The reaction mixture was dried on rota vapor at low pressure and then recrystallized with a 1:1 mixture of alcohol-chloroform to afford brownish crystals of the title compound (1.76 g, *M.p.* 406 K).

### Refinement

All H atoms were placed in their calculated positions, with C—H = 0.93 or 0.96 Å, and refined using a riding model, with *U*_iso_ = 1.2 or 1.5 *U*_eq_(C). The rotating group model is applied to the methyl groups.

### Crystal data

**Table:**

C_10_H_9_N_3_O_2_	*F*(000) = 848
*M**_r_* = 203.20	*D*_x_ = 1.437 Mg m^−^^3^
Monoclinic, *P*2_1_/*c*	Mo *K*α radiation, λ = 0.71073 Å
Hall symbol: -P 2ybc	Cell parameters from 9916 reflections
*a* = 7.1125 (7) Å	θ = 3.4–33.5°
*b* = 22.490 (2) Å	µ = 0.10 mm^−^^1^
*c* = 12.9596 (10) Å	*T* = 100 K
β = 115.026 (4)°	Block, brown
*V* = 1878.4 (3) Å^3^	0.26 × 0.21 × 0.10 mm
*Z* = 8	

### Data collection

**Table:**

Bruker APEXII DUO CCD diffractometer	7510 independent reflections
Radiation source: fine-focus sealed tube	5559 reflections with *I* > 2σ(*I*)
graphite	*R*_int_ = 0.043
φ and ω scans	θ_max_ = 33.8°, θ_min_ = 1.8°
Absorption correction: multi-scan (*SADABS*; Bruker, 2009)	*h* = −10→11
*T*_min_ = 0.973, *T*_max_ = 0.990	*k* = −35→35
52279 measured reflections	*l* = −20→20

### Refinement

**Table:**

Refinement on *F*^2^	Primary atom site location: structure-invariant direct methods
Least-squares matrix: full	Secondary atom site location: difference Fourier map
*R*[*F*^2^ > 2σ(*F*^2^)] = 0.045	Hydrogen site location: inferred from neighbouring sites
*wR*(*F*^2^) = 0.136	H-atom parameters constrained
*S* = 1.03	*w* = 1/[σ^2^(*F*_o_^2^) + (0.077*P*)^2^ + 0.2747*P*] where *P* = (*F*_o_^2^ + 2*F*_c_^2^)/3
7510 reflections	(Δ/σ)_max_ = 0.001
275 parameters	Δρ_max_ = 0.55 e Å^−^^3^
0 restraints	Δρ_min_ = −0.20 e Å^−^^3^

### Special details

**Table:**

Experimental. The crystal was placed in the cold stream of an Oxford Cryosystems Cobra open-flow nitrogen cryostat (Cosier & Glazer, 1986) operating at 100.0 (1)K.
Geometry. All esds (except the esd in the dihedral angle between two l.s. planes) are estimated using the full covariance matrix. The cell esds are taken into account individually in the estimation of esds in distances, angles and torsion angles; correlations between esds in cell parameters are only used when they are defined by crystal symmetry. An approximate (isotropic) treatment of cell esds is used for estimating esds involving l.s. planes.
Refinement. Refinement of F^2^ against ALL reflections. The weighted R-factor wR and goodness of fit S are based on F^2^, conventional R-factors R are based on F, with F set to zero for negative F^2^. The threshold expression of F^2^ > 2sigma(F^2^) is used only for calculating R-factors(gt) etc. and is not relevant to the choice of reflections for refinement. R-factors based on F^2^ are statistically about twice as large as those based on F, and R- factors based on ALL data will be even larger.

### Fractional atomic coordinates and isotropic or equivalent isotropic displacement parameters (Å^2^)

**Table:**

	*x*	*y*	*z*	*U*_iso_*/*U*_eq_	
O1A	0.42483 (15)	0.54231 (3)	0.13432 (7)	0.03320 (19)	
O2A	0.40149 (14)	0.57864 (4)	0.28272 (7)	0.03070 (18)	
N1A	0.46872 (13)	0.81176 (4)	0.03295 (7)	0.02043 (16)	
N2A	0.50093 (12)	0.79631 (4)	0.25642 (7)	0.01853 (15)	
N3A	0.41730 (13)	0.58434 (4)	0.19278 (7)	0.02205 (16)	
C1A	0.49512 (15)	0.85691 (4)	0.10172 (8)	0.02068 (17)	
C2A	0.45607 (13)	0.75638 (4)	0.07370 (8)	0.01702 (16)	
C3A	0.42621 (14)	0.70659 (4)	0.00252 (8)	0.01940 (17)	
H3A	0.4160	0.7116	−0.0709	0.023*	
C4A	0.41210 (14)	0.65075 (4)	0.04132 (8)	0.01985 (17)	
H4A	0.3915	0.6177	−0.0053	0.024*	
C5A	0.42934 (14)	0.64452 (4)	0.15257 (8)	0.01822 (16)	
C6A	0.45822 (14)	0.69133 (4)	0.22529 (8)	0.01792 (16)	
H6A	0.4686	0.6855	0.2985	0.022*	
C7A	0.47173 (13)	0.74866 (4)	0.18519 (7)	0.01652 (15)	
C8A	0.51266 (14)	0.84900 (4)	0.21625 (8)	0.01909 (16)	
C9A	0.54442 (18)	0.90164 (5)	0.29221 (9)	0.0257 (2)	
H9A	0.5650	0.8884	0.3667	0.039*	
H9B	0.4244	0.9269	0.2613	0.039*	
H9C	0.6642	0.9234	0.2975	0.039*	
C10A	0.5085 (2)	0.91790 (5)	0.05931 (10)	0.0309 (2)	
H10A	0.4890	0.9156	−0.0186	0.046*	
H10B	0.6425	0.9346	0.1049	0.046*	
H10C	0.4027	0.9426	0.0643	0.046*	
O1B	1.02498 (14)	0.88142 (4)	0.11899 (7)	0.03279 (18)	
O2B	1.01367 (14)	0.86371 (3)	0.28010 (7)	0.03235 (18)	
N1B	0.91353 (13)	0.60539 (4)	0.08423 (7)	0.02072 (15)	
N2B	0.96057 (12)	0.64557 (4)	0.30076 (7)	0.01937 (15)	
N3B	1.00834 (13)	0.84739 (4)	0.18868 (8)	0.02304 (17)	
C1B	0.91658 (15)	0.56851 (4)	0.16330 (8)	0.02119 (17)	
C2B	0.93380 (13)	0.66464 (4)	0.11081 (8)	0.01795 (16)	
C3B	0.92863 (15)	0.70603 (4)	0.02753 (8)	0.02017 (17)	
H3B	0.9091	0.6928	−0.0443	0.024*	
C4B	0.95218 (14)	0.76554 (4)	0.05198 (8)	0.02043 (17)	
H4B	0.9496	0.7930	−0.0022	0.025*	
C5B	0.98036 (14)	0.78392 (4)	0.16108 (8)	0.01887 (16)	
C6B	0.98399 (14)	0.74548 (4)	0.24403 (8)	0.01843 (16)	
H6B	1.0023	0.7595	0.3152	0.022*	
C7B	0.95944 (13)	0.68435 (4)	0.21894 (8)	0.01721 (16)	
C8B	0.93904 (15)	0.58899 (4)	0.27364 (8)	0.02046 (17)	
C9B	0.93881 (19)	0.54547 (5)	0.36108 (9)	0.0285 (2)	
H9D	0.9554	0.5664	0.4289	0.043*	
H9E	1.0512	0.5179	0.3786	0.043*	
H9F	0.8097	0.5242	0.3318	0.043*	
C10B	0.8977 (2)	0.50367 (5)	0.13618 (11)	0.0306 (2)	
H10D	0.8890	0.4978	0.0609	0.046*	
H10E	0.7747	0.4883	0.1399	0.046*	
H10F	1.0171	0.4832	0.1903	0.046*	

### Atomic displacement parameters (Å^2^)

**Table:**

	*U*^11^	*U*^22^	*U*^33^	*U*^12^	*U*^13^	*U*^23^
O1A	0.0509 (5)	0.0197 (3)	0.0321 (4)	−0.0016 (3)	0.0206 (4)	−0.0041 (3)
O2A	0.0432 (5)	0.0283 (4)	0.0244 (4)	−0.0055 (3)	0.0179 (3)	0.0018 (3)
N1A	0.0232 (4)	0.0210 (4)	0.0172 (4)	0.0026 (3)	0.0086 (3)	0.0016 (3)
N2A	0.0196 (3)	0.0201 (3)	0.0165 (3)	0.0004 (3)	0.0082 (3)	−0.0012 (3)
N3A	0.0244 (4)	0.0203 (4)	0.0210 (4)	−0.0024 (3)	0.0092 (3)	−0.0006 (3)
C1A	0.0230 (4)	0.0203 (4)	0.0185 (4)	0.0031 (3)	0.0085 (3)	0.0016 (3)
C2A	0.0161 (4)	0.0199 (4)	0.0152 (4)	0.0017 (3)	0.0067 (3)	0.0005 (3)
C3A	0.0208 (4)	0.0232 (4)	0.0153 (4)	0.0006 (3)	0.0086 (3)	−0.0019 (3)
C4A	0.0204 (4)	0.0216 (4)	0.0182 (4)	−0.0013 (3)	0.0088 (3)	−0.0033 (3)
C5A	0.0174 (4)	0.0188 (4)	0.0187 (4)	−0.0010 (3)	0.0079 (3)	−0.0003 (3)
C6A	0.0175 (4)	0.0208 (4)	0.0157 (4)	−0.0001 (3)	0.0073 (3)	−0.0004 (3)
C7A	0.0149 (3)	0.0201 (4)	0.0145 (4)	0.0004 (3)	0.0061 (3)	−0.0010 (3)
C8A	0.0195 (4)	0.0202 (4)	0.0178 (4)	0.0013 (3)	0.0081 (3)	−0.0011 (3)
C9A	0.0338 (5)	0.0209 (4)	0.0239 (5)	−0.0006 (4)	0.0137 (4)	−0.0044 (4)
C10A	0.0488 (7)	0.0204 (4)	0.0243 (5)	0.0037 (4)	0.0162 (5)	0.0043 (4)
O1B	0.0440 (5)	0.0202 (3)	0.0365 (5)	−0.0034 (3)	0.0192 (4)	0.0045 (3)
O2B	0.0469 (5)	0.0206 (3)	0.0330 (4)	−0.0026 (3)	0.0203 (4)	−0.0061 (3)
N1B	0.0221 (4)	0.0186 (3)	0.0209 (4)	−0.0005 (3)	0.0085 (3)	−0.0020 (3)
N2B	0.0200 (3)	0.0183 (3)	0.0195 (4)	0.0003 (3)	0.0081 (3)	0.0010 (3)
N3B	0.0229 (4)	0.0176 (3)	0.0284 (4)	−0.0011 (3)	0.0106 (3)	−0.0001 (3)
C1B	0.0223 (4)	0.0171 (4)	0.0234 (4)	0.0008 (3)	0.0089 (3)	−0.0009 (3)
C2B	0.0159 (4)	0.0186 (4)	0.0187 (4)	−0.0005 (3)	0.0066 (3)	−0.0011 (3)
C3B	0.0208 (4)	0.0212 (4)	0.0188 (4)	−0.0017 (3)	0.0086 (3)	−0.0003 (3)
C4B	0.0194 (4)	0.0207 (4)	0.0213 (4)	−0.0007 (3)	0.0087 (3)	0.0018 (3)
C5B	0.0174 (4)	0.0160 (4)	0.0233 (4)	−0.0009 (3)	0.0087 (3)	−0.0006 (3)
C6B	0.0180 (4)	0.0181 (4)	0.0198 (4)	−0.0009 (3)	0.0085 (3)	−0.0017 (3)
C7B	0.0157 (3)	0.0173 (4)	0.0186 (4)	−0.0006 (3)	0.0072 (3)	−0.0010 (3)
C8B	0.0206 (4)	0.0190 (4)	0.0209 (4)	0.0009 (3)	0.0079 (3)	0.0014 (3)
C9B	0.0386 (6)	0.0209 (4)	0.0255 (5)	−0.0002 (4)	0.0131 (4)	0.0042 (4)
C10B	0.0433 (6)	0.0176 (4)	0.0334 (6)	−0.0009 (4)	0.0188 (5)	−0.0027 (4)

### Geometric parameters (Å, °)

**Table:**

O1A—N3A	1.2267 (11)	O1B—N3B	1.2272 (11)
O2A—N3A	1.2242 (11)	O2B—N3B	1.2255 (12)
N1A—C1A	1.3111 (12)	N1B—C1B	1.3114 (12)
N1A—C2A	1.3705 (12)	N1B—C2B	1.3686 (12)
N2A—C8A	1.3111 (12)	N2B—C8B	1.3117 (12)
N2A—C7A	1.3717 (11)	N2B—C7B	1.3703 (12)
N3A—C5A	1.4658 (12)	N3B—C5B	1.4646 (12)
C1A—C8A	1.4469 (13)	C1B—C8B	1.4454 (14)
C1A—C10A	1.4956 (14)	C1B—C10B	1.4927 (14)
C2A—C3A	1.4087 (13)	C2B—C7B	1.4062 (13)
C2A—C7A	1.4121 (12)	C2B—C3B	1.4138 (13)
C3A—C4A	1.3722 (13)	C3B—C4B	1.3693 (13)
C3A—H3A	0.9300	C3B—H3B	0.9300
C4A—C5A	1.4014 (13)	C4B—C5B	1.4036 (13)
C4A—H4A	0.9300	C4B—H4B	0.9300
C5A—C6A	1.3691 (13)	C5B—C6B	1.3711 (13)
C6A—C7A	1.4086 (13)	C6B—C7B	1.4066 (12)
C6A—H6A	0.9300	C6B—H6B	0.9300
C8A—C9A	1.4944 (13)	C8B—C9B	1.4978 (14)
C9A—H9A	0.9600	C9B—H9D	0.9600
C9A—H9B	0.9600	C9B—H9E	0.9600
C9A—H9C	0.9600	C9B—H9F	0.9600
C10A—H10A	0.9600	C10B—H10D	0.9600
C10A—H10B	0.9600	C10B—H10E	0.9600
C10A—H10C	0.9600	C10B—H10F	0.9600
			
C1A—N1A—C2A	117.11 (8)	C1B—N1B—C2B	117.01 (8)
C8A—N2A—C7A	117.13 (8)	C8B—N2B—C7B	116.63 (8)
O2A—N3A—O1A	123.57 (9)	O2B—N3B—O1B	123.47 (9)
O2A—N3A—C5A	118.55 (8)	O2B—N3B—C5B	118.27 (8)
O1A—N3A—C5A	117.88 (8)	O1B—N3B—C5B	118.27 (9)
N1A—C1A—C8A	121.84 (9)	N1B—C1B—C8B	121.99 (9)
N1A—C1A—C10A	118.27 (8)	N1B—C1B—C10B	117.64 (9)
C8A—C1A—C10A	119.90 (9)	C8B—C1B—C10B	120.37 (9)
N1A—C2A—C3A	119.10 (8)	N1B—C2B—C7B	120.87 (8)
N1A—C2A—C7A	121.09 (8)	N1B—C2B—C3B	118.88 (8)
C3A—C2A—C7A	119.80 (8)	C7B—C2B—C3B	120.25 (8)
C4A—C3A—C2A	120.12 (8)	C4B—C3B—C2B	120.38 (9)
C4A—C3A—H3A	119.9	C4B—C3B—H3B	119.8
C2A—C3A—H3A	119.9	C2B—C3B—H3B	119.8
C3A—C4A—C5A	118.70 (8)	C3B—C4B—C5B	118.18 (9)
C3A—C4A—H4A	120.6	C3B—C4B—H4B	120.9
C5A—C4A—H4A	120.6	C5B—C4B—H4B	120.9
C6A—C5A—C4A	123.62 (8)	C6B—C5B—C4B	123.47 (9)
C6A—C5A—N3A	118.66 (8)	C6B—C5B—N3B	117.86 (8)
C4A—C5A—N3A	117.72 (8)	C4B—C5B—N3B	118.67 (8)
C5A—C6A—C7A	117.63 (8)	C5B—C6B—C7B	118.40 (9)
C5A—C6A—H6A	121.2	C5B—C6B—H6B	120.8
C7A—C6A—H6A	121.2	C7B—C6B—H6B	120.8
N2A—C7A—C6A	118.79 (8)	N2B—C7B—C2B	121.76 (8)
N2A—C7A—C2A	121.09 (8)	N2B—C7B—C6B	118.93 (8)
C6A—C7A—C2A	120.12 (8)	C2B—C7B—C6B	119.31 (8)
N2A—C8A—C1A	121.74 (8)	N2B—C8B—C1B	121.72 (9)
N2A—C8A—C9A	118.14 (8)	N2B—C8B—C9B	117.94 (9)
C1A—C8A—C9A	120.12 (8)	C1B—C8B—C9B	120.34 (9)
C8A—C9A—H9A	109.5	C8B—C9B—H9D	109.5
C8A—C9A—H9B	109.5	C8B—C9B—H9E	109.5
H9A—C9A—H9B	109.5	H9D—C9B—H9E	109.5
C8A—C9A—H9C	109.5	C8B—C9B—H9F	109.5
H9A—C9A—H9C	109.5	H9D—C9B—H9F	109.5
H9B—C9A—H9C	109.5	H9E—C9B—H9F	109.5
C1A—C10A—H10A	109.5	C1B—C10B—H10D	109.5
C1A—C10A—H10B	109.5	C1B—C10B—H10E	109.5
H10A—C10A—H10B	109.5	H10D—C10B—H10E	109.5
C1A—C10A—H10C	109.5	C1B—C10B—H10F	109.5
H10A—C10A—H10C	109.5	H10D—C10B—H10F	109.5
H10B—C10A—H10C	109.5	H10E—C10B—H10F	109.5
			
C2A—N1A—C1A—C8A	0.68 (14)	C2B—N1B—C1B—C8B	0.35 (14)
C2A—N1A—C1A—C10A	−179.74 (9)	C2B—N1B—C1B—C10B	−179.15 (9)
C1A—N1A—C2A—C3A	179.54 (9)	C1B—N1B—C2B—C7B	0.83 (13)
C1A—N1A—C2A—C7A	−0.23 (13)	C1B—N1B—C2B—C3B	−179.19 (9)
N1A—C2A—C3A—C4A	−179.69 (8)	N1B—C2B—C3B—C4B	−178.82 (9)
C7A—C2A—C3A—C4A	0.09 (13)	C7B—C2B—C3B—C4B	1.16 (14)
C2A—C3A—C4A—C5A	−0.42 (14)	C2B—C3B—C4B—C5B	−0.29 (14)
C3A—C4A—C5A—C6A	0.49 (14)	C3B—C4B—C5B—C6B	−0.49 (14)
C3A—C4A—C5A—N3A	−178.99 (8)	C3B—C4B—C5B—N3B	179.24 (8)
O2A—N3A—C5A—C6A	11.04 (13)	O2B—N3B—C5B—C6B	−6.86 (13)
O1A—N3A—C5A—C6A	−168.57 (9)	O1B—N3B—C5B—C6B	173.21 (9)
O2A—N3A—C5A—C4A	−169.46 (9)	O2B—N3B—C5B—C4B	173.39 (9)
O1A—N3A—C5A—C4A	10.93 (13)	O1B—N3B—C5B—C4B	−6.54 (13)
C4A—C5A—C6A—C7A	−0.20 (14)	C4B—C5B—C6B—C7B	0.38 (14)
N3A—C5A—C6A—C7A	179.27 (8)	N3B—C5B—C6B—C7B	−179.35 (8)
C8A—N2A—C7A—C6A	−179.96 (8)	C8B—N2B—C7B—C2B	0.80 (13)
C8A—N2A—C7A—C2A	0.28 (13)	C8B—N2B—C7B—C6B	−179.40 (8)
C5A—C6A—C7A—N2A	−179.91 (8)	N1B—C2B—C7B—N2B	−1.47 (13)
C5A—C6A—C7A—C2A	−0.15 (13)	C3B—C2B—C7B—N2B	178.54 (8)
N1A—C2A—C7A—N2A	−0.27 (13)	N1B—C2B—C7B—C6B	178.72 (8)
C3A—C2A—C7A—N2A	179.96 (8)	C3B—C2B—C7B—C6B	−1.26 (13)
N1A—C2A—C7A—C6A	179.98 (8)	C5B—C6B—C7B—N2B	−179.31 (8)
C3A—C2A—C7A—C6A	0.21 (13)	C5B—C6B—C7B—C2B	0.50 (13)
C7A—N2A—C8A—C1A	0.16 (13)	C7B—N2B—C8B—C1B	0.39 (13)
C7A—N2A—C8A—C9A	179.99 (8)	C7B—N2B—C8B—C9B	−179.77 (9)
N1A—C1A—C8A—N2A	−0.68 (15)	N1B—C1B—C8B—N2B	−1.02 (15)
C10A—C1A—C8A—N2A	179.74 (9)	C10B—C1B—C8B—N2B	178.47 (9)
N1A—C1A—C8A—C9A	179.49 (9)	N1B—C1B—C8B—C9B	179.14 (9)
C10A—C1A—C8A—C9A	−0.08 (14)	C10B—C1B—C8B—C9B	−1.37 (15)

### Hydrogen-bond geometry (Å, °)

**Table:**

*D*—H···*A*	*D*—H	H···*A*	*D*···*A*	*D*—H···*A*
C3A—H3A···N2A^i^	0.93	2.56	3.4486 (14)	160
C9B—H9D···O1B^ii^	0.96	2.58	3.5380 (14)	176
C10A—H10A···O2A^i^	0.96	2.38	3.3355 (15)	171

Symmetry codes: (i) *x*, −*y*+3/2, *z*−1/2; (ii) *x*, −*y*+3/2, *z*+1/2.
